# Simultaneous Implant and Guided Bone Regeneration Using Bovine-Derived Xenograft and Acellular Dermal Matrix in Aesthetic Zone

**DOI:** 10.3390/dj12030052

**Published:** 2024-02-26

**Authors:** Anggun Alfreda Devina, Felita Clarissa Halim, Benso Sulijaya, Patricia Rinanti Sumaringsih, Ratna Sari Dewi

**Affiliations:** 1 Periodontology Specialist Program, Department of Periodontology, Faculty of Dentistry, Universitas Indonesia, Jakarta 10430, Indonesia; anggunalfreda@gmail.com (A.A.D.); felita_clarissa@hotmail.com (F.C.H.); 2 Department of Periodontology, Faculty of Dentistry, Universitas Indonesia, Jakarta 10430, Indonesia; 3 Prosthodontics Specialist Program, Department of Prosthodontics, Faculty of Dentistry, Universitas Indonesia, Jakarta 10430, Indonesia; drg.patriciarinanti@gmail.com; 4 Department of Prosthodontics, Faculty of Dentistry, Universitas Indonesia, Jakarta 10430, Indonesia; ratnasaridewi.drg@gmail.com

**Keywords:** guided bone regeneration, dental implants, xenograft, acellular dermal matrix, dentistry, oral surgery

## Abstract

Introduction: Implant placement in the maxillary anterior area requires sufficient quantity and quality of both soft and hard tissue. In cases where soft and hard tissues are insufficient, additional regeneration using biomaterials is recommended. Treatment using bovine-derived xenograft and acellular dermal matrix (ADM) may increase bone volume and soft tissue thickness. Case and management: A 65-year-old woman sought help for discomfort and aesthetic issues with her denture, reporting missing teeth (11, 12, 13, 14, and 21) and bone volume shrinkage due to disuse atrophy. Intraoral examination revealed 1 mm gingival thickness. CBCT showed labio-palatal bone thickness of 6.0 mm, 5.8 mm, and 4.7 mm for teeth 21, 12 and 14, respectively. Implant planning and surgical guide fabrication were carried out before the surgery. Surgery included the placement of implants 3.3 mm in diameter and 12 mm in length, with the use of xenograft and ADM. Three months post-op, improvements in soft and hard tissues were observed, with a final prosthesis being a long-span implant-supported bridge. Conclusions: Disuse alveolar atrophy causes soft and hard tissue deficiency. The use of xenograft and ADM show favourable results even on a geriatric patient.

## 1. Introduction

Guided Bone Regeneration (GBR) is a fundamental technique within the realm of dentistry employed for the restoration and replacement of compromised bone in the peri-tooth or peri-implant regions. This intricate approach aims to orchestrate guided bone growth to create an optimal environment conducive to subsequent dental implant placement or restoration of lost tooth function [1]. Often integrated with implant procedures, GBR becomes particularly crucial in addressing volumetric changes in both maxillary and mandibular bones post-tooth loss. These changes, occurring in the range of 29% to 63% in horizontal and intermediate dimensions, pose substantial impediments to effective dental implant rehabilitation. The clinical manifestation of GBR faces distinct challenges, notably in regions exhibiting vertical bone damage, with volume reductions ranging from 11% to 22% within six months post-tooth loss. Addressing these challenges is paramount for the successful implementation of GBR across diverse clinical contexts, underlining its pivotal role in contemporary dental practices [2,3].

Various techniques and biomaterials have been used to address this discrepancy in bone grafting. These materials are classified, based on their origin, as autogenous, xenogenous, allogenous and alloplastic. Autogenous bone grafts possess osteogenic, osteo-inductive, and osteo-conductive properties, but their use entails surgical procedures in both donor and recipient areas, increasing the risk of morbidity [3,4,5]. In contrast, synthetic grafts, whether homogeneous or heterogeneous, exhibit primarily osteo-conductive properties. When combined with autogenous bone or growth factors, these materials serve as a framework, facilitating the adhesion and proliferation of osteoprogenitor cells. This comprehensive understanding of grafting materials and techniques is crucial for optimizing outcomes in bone augmentation procedures [2,5].

The foundational principle of GBR, encapsulated in the acronym P.A.S.S (Primary closure, Angiogenesis, Space maintenance, and Stability of wound and implant), is fundamental for the accurate prediction of bone regeneration outcomes. Ensuring primary wound closure is imperative to facilitate uninterrupted wound healing. Angiogenesis plays a crucial role in providing essential blood supply and undifferentiated mesenchymal cells. Space maintenance is of the utmost importance to create an environment conducive to optimal bone growth. Simultaneously, wound and implant stability are integral factors in promoting blood clot formation and fostering a robust healing process. This comprehensive adherence to the P.A.S.S principles underscores their significance in orchestrating successful bone regeneration and serves as a guiding framework for practitioners in the realm of guided bone regeneration procedures [5,6].

The application of the GBR technique to the maxillary anterior teeth is crucial, given the intricate considerations of bone structure, biological characteristics, aesthetic demands, and procedural complexity [6,7]. In achieving optimal outcomes, careful attention must be paid to existing morphological factors, coupled with the prudent selection of appropriate materials [8]. The sustained stability of both soft and hard tissues surrounding the implant emerges as a critical determinant for the long-term success of implant treatments. Within the biological context, parameters such as keratinized mucosa width, attached mucosa, mucosal thickness, supra-crestal soft tissue height, and osseointegration significantly influence implant performance [9]. Farronato et al. advocate for maintaining a minimum buccal bone thickness of 1.5 mm to mitigate the risk of gingival margin recession, particularly in aesthetic regions [10]. Similarly, Ortiz et al. emphasize a minimum mucosal tissue thickness of 2 mm to prevent the shadow effect of the implant abutment, gingival recession, and buccal plate depression [9]. Implant installation in the aesthetic zone necessitates meticulous attention to the position, angulation, and distances between buccal and palatal implants and surrounding teeth, as well as the inter-implant spacing, all aimed at minimizing potential structural damage and averting bone resorption around the implant. This comprehensive approach underscores the intricate interplay of factors crucial for achieving successful outcomes in aesthetic zone implantology [11].

Acellular Dermal Matrix (ADM) is typically used for soft tissue augmentation and as an alternative to the subepithelial connective tissue graft for mucogingival procedures [12,13]. De Andrade et al. validated the capability of ADM to function as a membrane in Guided Tissue Regeneration (GTR) procedures for grade 2 furcation defects [14]. The capacity of ADM also plays important role in maintaining tissue architecture in the healing phase [13,14]. There are limited studies regarding the use of ADM as a membrane for GBR. In animal studies, there were no statistically significant differences between the groups 16 weeks after employing GBR with ADM as a barrier membrane compared to the traditional collagen membrane without a bone graft [15].

The primary objective of this case report is to evaluate the effectiveness of GBR and soft tissue thickness augmentation in the placement of dental implants in the maxillary anterior region. The bone augmentation procedure aims to address and correct bone defects, creating favorable dimensions for the successful placement of dental implants.

## 2. Case Report

A 65-year-old female patient sought dental consultation due to discomfort associated with removable dentures and the absence of anterior teeth, impacting both aesthetic appearance and masticatory function. The UCLA classification categorized the remaining alveolar bone as class 2 [16]. Intraoral examination revealed the absence of maxillary anterior teeth, inadequate mucosal tissue thickness, and diminished bone volume attributed to disuse atrophy [17]. Despite having received artificial teeth for both upper and lower jaws, the patient opted not to use them, citing persistent feelings of nausea and discomfort. This case underscores the multifaceted challenges in prosthodontic management, emphasizing the need for a comprehensive treatment approach addressing both functional and patient-centered considerations.

**Figure 1:**
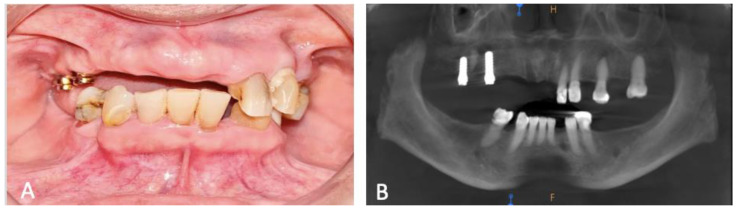
Pre-operative assessment (**A**) Clinical appearance of anterior maxilla at baseline. (**B**) Orthopantomagram (OPG). H stands for height and F stands for floor indication superior and inferior border of image.

Prior to initiating any treatment, the patient provided informed consent, indicating their agreement for the proposed procedures. In preparation for the intervention, intraoral photos were taken, complemented by panoramic radiographs and a 3D cone-beam computed tomography (CBCT) scan (Figure 1A,B). The CBCT findings revealed labio-palatal ridge dimensions of 15.33 mm, 6.23 mm, and 8.86 mm for teeth 14, 12, and 21, respectively. A study model was then created to formulate the therapeutic plan. Radiographically, the results indicated lower bone density and thin bone volume. Consequently, the treatment plan for this case involves the placement of dental implants.

The presence of bone defects in this area necessitates a bone augmentation procedure, with the primary choice being the use of bone replacement material in the form of a bone graft [18]. Additionally, other examinations, including an assessment of soft tissue availability, are taken into account. Comprehensive measurements of the width of the attached gingiva, vestibule, and soft tissue thickness were conducted to establish clinical parameters as references for subsequent surgical procedures [19]. Factors such as oral hygiene conditions and ensuring the absence of systemic conditions that may impact treatment planning are also considered, as they contribute to the overall success of the treatment [20,21].

Based on a thorough assessment of relevant clinical parameters, the proposed treatment plan involves the simultaneous installation of implants in the maxillary anterior teeth, complemented by the addition of xenograft mixed with autogenous bone graft and the strategic utilization of ADM as a barrier to enhance mucosal tissue thickness [22,23]. The patient’s decade-long history of successfully installed implants in teeth 15 and 17 facilitates the acceptance and feasibility of additional implants. Implant location, type, and size were determined through comprehensive planning using 3Shape software, (Implant Studio version 2020.1.0) which involved superimposing CBCT radiographs (Vatech, Gyeonggi-do, South Korea) and study model scan results. In the anterior maxilla, a 5-unit bone-level implant bridge with screw-retained abutments will be crafted for regions 14, 12, and 21, while modified ridge lap-type pontics will be used in regions 13 and 11, utilizing monolithic zirconia material. The surgical approach to soft tissue involves using a trapezoid full-thickness mucoperiosteal flap design on teeth 14, 12, and 21 [24]. This design, meticulously crafted, ensures optimal accessibility and visibility during the surgical procedure [25]. The selection of scalpel No. 15c is deemed advantageous for making incisions, given the irregular position and architecture of the edentulous area [26,27]. Administering local anesthesia with vasoconstrictor agents is instrumental in prolonging the duration of action and mitigating the risk of bleeding [5].

**Figure 2:**
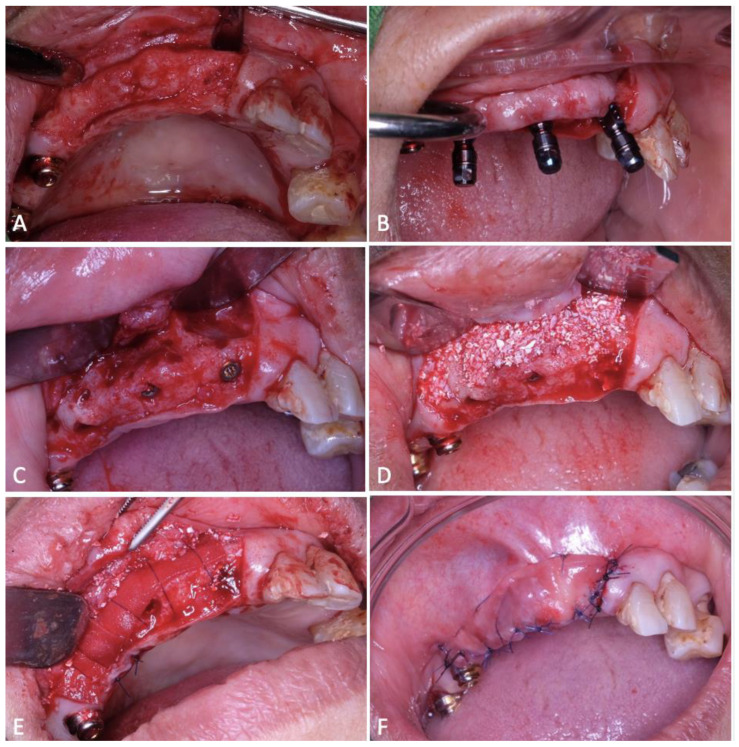
Surgical procedure (**A**) Opening of the trapezoidal full-thickness muco-periosteal flap. (**B**) Placement of implants on teeth 14, 12, 21. (**C**) Decortication to obtain vascularization. (**D**) Placement of bovine-derived xenograft mix with autogenous graft. (**E**) Placement of the membrane stabilized by suturing. (**F**) Suturing with vertical internal mattresses, continuous locking, and interrupted suture.

After the precise opening of the flap, the releasing flap procedure is systematically carried out before implant placement, aiming for optimal closure after GBR and implant placement (Figure 2A) [5,24]. The meticulous removal of all granulation tissue was followed by saline irrigation to prevent inflammation [28]. Implant placement, conducted by a prosthodontics department resident, occurred in stages, initiating with teeth 14, 12, and 21, utilizing implants with a diameter of 3.3 mm and a length of 12 mm (BLT Implants, Straumann, Basel, Switzerland) (Figure 2B).

Following the attainment of primary stability, decortication was performed around the bone to stimulate additional intraosseous vascularization, thereby promoting the angiogenesis process (Figure 2C) [29]. Afterwards, the bone defect area received a bovine-derived xenograft (Ti-oss, Chiyewon, Republic of Korea) combined with autografts taken during implant oseteotomy and extracted from the buccal bone area using a scraper (Figure 2D) [20] (Figure 2D) [22,30]. Subsequently, the ADM was meticulously positioned to secure the bone graft in place, and the matrix was fixated using resorbable sutures (Luxcryl PDO, Lux Sutures, Luxembourg) (Figure 2E). The incorporation of ADM in this augmentation aimed to augment soft tissue thickness while acting as a barrier to impede soft tissue migration towards the bone graft [23,31]. Suturing was meticulously performed using non-resorbable 5-0 nylon (Luxamid, Lux Sutures, Luxembourg) employing a vertical internal mattress suture technique, combined with continuous locking sutures and simple interrupted sutures (Figure 2F) [24]. The patient received a 5-day prescription of Amoxicillin-clavulanate antibiotic, anti-inflammatory drugs, and a chlorhexidine gluconate 0.2% antiseptic mouthwash to forestall infection or secondary inflammation.

**Figure 3:**
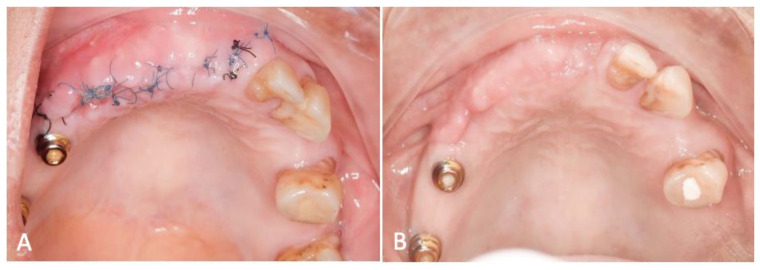
Follow-up evaluation (**A**) at 2 weeks and (**B**) 4 weeks post-op.

The patient’s post-procedural assessment, conducted two weeks after the intervention, revealed that the gingival condition had not reached complete resolution, displaying residual minor inflammation (Figure 3A). During this follow-up, all sutures were meticulously removed, and the patient received thorough instructions on maintaining optimal oral hygiene. Subsequent evaluations at the 4th week (Figure 3B) and 12th week demonstrated marked clinical improvement, with the complete subsidence of gingival inflammation.

**Figure 4:**
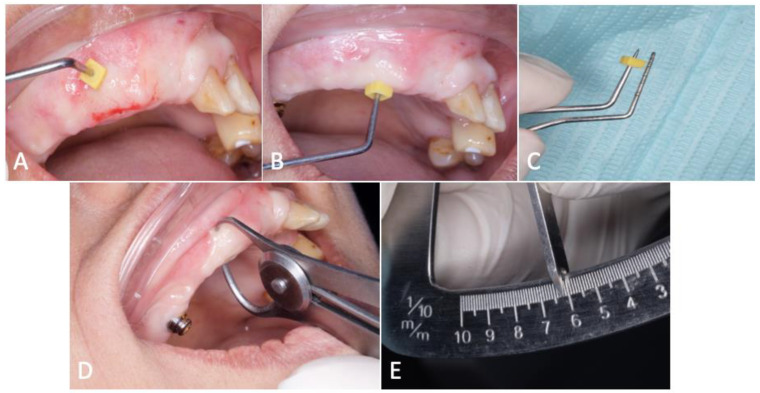
Three-month post-op reassessment. (**A**–**D**) Soft tissue thickness measurements were conducted through a series of assessments using an endodontic stopper and a straight probe, (**E**) while bone thickness was measured using a bone caliper.

At the 3-month post-op, both tissues exhibited visible thickening, indicative of stability. Mucosal thickness measurements were conducted using a specialized tool, the UNC-15 probe (Hu-Friedy, Chicago, IL, USA), incorporating a rubber stopper. Measurements involved precise insertion until the probe reached the bone, with subsequent calculation of the distance between the probe’s tip and the stopper. Thickness assessments were performed on the buccal, occlusal, and palatal aspects. Additionally, anterior bone thickness measurements were conducted using a bone caliper (Figure 4A–E). Complementary to these assessments, a CBCT radiographic examination provided a comprehensive evaluation of the bone condition surrounding the implant. Follow-up evaluation consistently demonstrated favorable and progressive healing outcomes.

**Figure 5:**
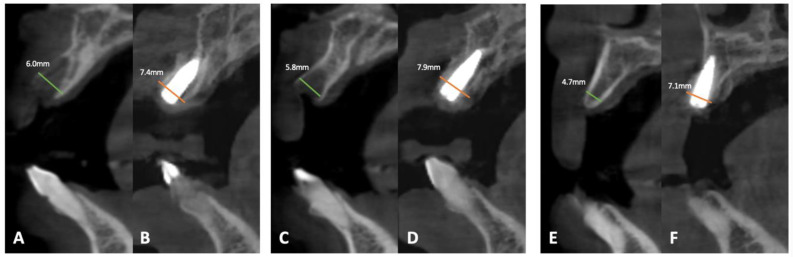
Four-month post-op CBCT evaluation of (**A**,**B**) tooth 21, (**C**,**D**) tooth 12, and (**E**,**F**) tooth 14.

In this case, a notable increase in soft tissue thickness was observed, progressing from 1 mm to 2 mm within 4 months. Additionally, as depicted in Figure 5, there was a marked augmentation in peri-implant bone volume surrounding teeth 21, 12, and 14, elevating from 6.0 mm, 5.8 mm, and 4.7 mm to 7.4 mm, 7.9 mm, and 7.4 mm, respectively, after the same duration (Table 1). These findings underscore the efficacy of the GBR using ADM, demonstrating favorable outcomes not only functionally but also aesthetically.

The second-stage surgery was conducted six months following the initial implant placements, employing a Straumann NC healing abutment with a diameter of 3.6 mm and a height of 5 mm. Subsequent to the placement of the healing abutment, a double impression was taken using the open-tray technique after one month. Before the delivery of the anterior long-span implant bridge, it is important to highlight that the patient had previously undergone posterior prosthesis placement, ensuring the establishment of occlusal support.

**Figure 6:**
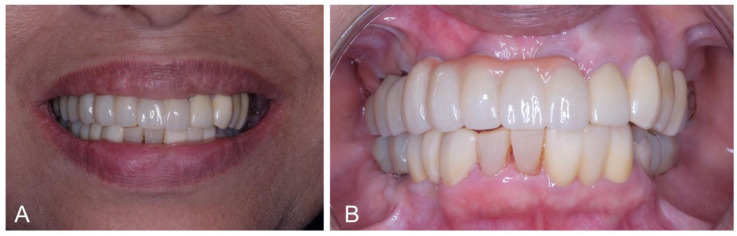
7-month post-op clinical photograph of final prosthesis delivery (**A**,**B**).

The preference for a cement-retained abutment in this case stems from its ability to enhance aesthetics by eliminating the need for screw access holes, thereby achieving a natural appearance akin to original teeth [32]. This becomes particularly significant given the specific case scenario where the screw access hole is positioned labially on tooth 21. Opting for zirconia as both the restorative material and abutment further contributes to elevated dental and gingival aesthetics in implant restorations. Zirconia’s commendable attributes, including excellent biocompatibility, high flexural strength, and toughness, make it an ideal choice. Notably, the zirconia abutment prevents the manifestation of a grayish hue in peri-implant gingival tissue, resulting in enhanced translucency and a brighter gingival presentation [33,34,35]. The fabrication of these abutments can be tailored using advanced CAD/CAM technology or achieved through prefabrication with subsequent modification via conventional preparation techniques [36]. Final prosthesis delivery is as seen on Figure 6.

Patients were thoroughly instructed on the importance of maintaining cleanliness around the prosthesis and underlying tissues. They were advised to utilize a soft toothbrush with proper technique and a water irrigator to effectively remove any food remnants. Additionally, emphasizing the significance of regular check-ups every 3 to 6 months was crucial. These appointments facilitate the evaluation of the prosthesis and peri-implant tissues, allowing for timely assessment and professional cleaning when necessary.

## 3. Discussion

The success of implant placement relies on the availability of adequate bone. In edentulous areas, bone dimensions undergo changes due to the resorption process, making the selection of the appropriate implant diameter and length challenging, especially when in proximity to anatomical structures [37,38,39]. In the presented case, the patient underwent anterior tooth extraction approximately 6 months before the implant placement. A nuanced understanding of the biological processes governing bone and soft tissue changes post-extraction is crucial to achieve satisfactory functional and aesthetic results. Thin buccal bone plates in extracted teeth often undergo horizontal and vertical resorption, particularly within the initial 6 to 12 months post-extraction [40]. This bone remodeling occurs in two phases, with initial resorption during the first 3 months followed by new bone formation in the subsequent 3 months. However, the alveolar bone height and width are reduced by approximately two-thirds of their original dimensions. Between months 6 to 12, continued bone remodeling leads to a further decrease, potentially reaching 50% of the initial dimensions [41]. When faced with insufficient bone volume, graft materials become imperative to achieve the correct dimensions for implant placement, ensuring stability and optimal healing [1,42].

During the implant placement, although there was no evident dehiscence or buccal fenestration, a bone graft was incorporated due to the acknowledged significance of buccal bone thickness (BBT) in ensuring the long-term stability of the implant. A prospective study conducted by Farronato et al. emphasized the critical role of buccal bone thickness at the time of implant placement in determining the stability of the gingival turnover over an extended period [10]. Their data analysis demonstrated a robust correlation between BBT and the tendency of buccal gums to reposition either coronally or apically during a maturation period of three years. These findings align with Cicciu et al., who highlighted a noteworthy association between BBT and the vertical stability of the buccal bone [43]. Specifically, when BBT was less than 1.8 mm, vertical buccal bone resorption occurred, whereas BBT greater than or equal to 1.8 mm indicated more predictable bone stability in subsequent operations [15]. Furthermore, BBT extends its impact to the peri-implant soft tissue. A BBT range of >0.5 mm to <1.5 mm was correlated with buccal gingival recession measuring 0.64 ± 0.29 mm, while a BBT value of ≥1.5 mm corresponded to a coronal growth of the gingiva by 0.77 ± 0.22 mm [10]. In cases of absent or deficient BBT, Buser et al. advocate for volumetric augmentation using surgical techniques such as GBR [44].

A xenograft, derived from a different species than the recipient, serves as a graft material produced by deproteinating organic material from the bones of cattle, horses, and pigs [45]. This method results in a porous structure akin to cancellous bone in humans, offering high osteoconduction due to its gradual and beneficial absorption that defends the space [46]. The absorption of xenografts, particularly deproteinized bovine bone mineral (DBBM), plays a crucial role in maintaining the volume of space, as excessive absorption may delay the formation of new bone [30]. To address this concern, researchers have focused on developing materials with high biocompatibility that mirror the remodeling characteristics of human bones. Octacalcium phosphate (OCP), known for its biodegradable properties, is applied as a coating on Ti-oss^®^ surfaces, assumed to be biological apatite precursors of dentin, enamel, and bone crystals [47]. The chemical degradation and biodegradation of OCP by osteoclast-like cells occur in a physiological environment, inducing osteoclast cells and expediting new bone formation, similar to autogenous materials [30]. The versatility of OCP proves advantageous in cases of extensive bone loss or when large volumes of bone are required [5,48]. Studies, such as the one by Temmerman et al., have demonstrated that the use of pure xenografts, even in conditions of bone defects, can yield satisfactory results [49]. The placement of membranes as mechanical barriers serves to separate the surgical site from epithelial cells and connective tissue, thereby creating favorable conditions for osteogenic cell proliferation between the 3rd and 12th week, facilitating subsequent bone formation. Typically, these membranes are positioned over the grafting material to uphold space and stability for the fibrin clot formed within the surgical area [48].

The soft tissue biotype plays a crucial role in achieving optimal aesthetic outcomes and promoting the success of implant restorations while minimizing the risk of future mucosal recession [50]. In the presented case, the initial soft tissue thickness before implant placement measured only 1 mm, categorizing it as a thin soft tissue biotype according to the classification by Linkevicius et al. [51]. The chosen implant design incorporates a platform-switching connection between the implant and the abutment. Research by Canullo et al. has highlighted the superiority of platform-switched implants in maintaining bone stability in crystalline bone [52]. Addressing the interaction between platform-switching and soft tissue biotype, Linkevicius et al. reported notable outcomes. In cases where platform-switching is employed with thin soft tissue, bone resorption measured 0.79 mm after 2 months and 1.17 mm after 1 year. Conversely, with a thick soft tissue biotype, platform-switching implants demonstrated the ability to preserve crestal height of alveolar bone with minimal remodeling, registering at 0.12 mm after 2 months and 0.21 mm after 1 year [51].

Acellular Dermal Matrix serves as a valuable resource for augmenting soft tissue thickness [31]. In addition to its availability in substantial quantities, ADM proves advantageous for cases necessitating a multitude of grafts to address wide defects, eliminating the need for donor site surgery as required for procedures like connective tissue grafts (CTG) [53,54]. The utilization of ADM in enhancing peri-implant soft tissue thickness is documented in the research conducted by Puisys et al. Prior to the procedure, the soft tissue exhibited an average thickness of 1.54 ± 0.51 mm. Following implant placement and the application of ADM, the thickness significantly increased to 3.75 ± 0.54 mm after a span of 3 months. It is noteworthy, however, that this study did not incorporate the use of bone grafts in conjunction with ADM [54].

The utilization of ADM as a membrane in combination with xenograft is not extensively documented in existing literature. To the best of the authors’ knowledge, only four studies have explored this specific combination, with one conducted in animal studies and three in humans [15,55,56,57]. Notably, all these studies have collectively demonstrated the efficacy of ADM as a barrier membrane in GBR, evidenced by favorable bone maturation upon re-entry. ADM, in this context, serves a dual function, as elucidated in various studies, functioning both as a barrier membrane for additional bone and as a means to augment soft tissue thickness [55,57]. However, it is noteworthy that in animal studies, no significant difference was observed between the use of ADM and collagen membranes in the augmentation of keratinized tissue when employed for GBR [15]. In our presented case report, a 3-month follow-up revealed apparent thickening of soft tissue from approximately 1 mm to 2 mm. Despite these promising findings, the use of ADM as a barrier membrane necessitates further comprehensive studies to ascertain its true dual functionality in GBR treatment.

The stability of the outcomes achieved through the present technique is intricately linked to adherence to proper oral hygiene protocols [58] and the necessity for long-term evaluation [59]. This endeavor necessitates the guidance and support of dental professionals in assisting patients with their home care routines, taking into careful consideration individual motivation levels and the efficacy of hygiene procedures. Regular check-ups are essential, involving an assessment of the restoration and nearby tissues. These appointments should encompass a meticulous evaluation of the restoration’s condition and the health of surrounding tissues. Alongside this assessment, comprehensive professional cleaning of prostheses, implants, and abutments should be conducted [60].

In this case report, several factors restrict the overall procedure. The surgical operation results in significant injuries in the operating area. Another limiting factor in optimizing treatment time is the extended waiting period for patients to receive the final prosthesis. The final factor is the recent placement of the prosthesis, hindering the follow-up of the post-prosthesis tissue maintenance phase.

## 4. Conclusions

In conclusion, addressing inadequate buccal bone through GBR in dental implant placement stands as a pivotal strategy for ensuring the enduring success of implant treatments. The incorporation of xenografts with ADM as a barrier membrane in GBR emerges as a promising approach to enhance both peri-implant hard and soft tissue volumetrics. This comprehensive technique not only offers significant advantages in terms of function, long-term stability, and aesthetics but also holds the potential to contribute positively to the overall well-being and satisfaction of patients undergoing implant procedures, particularly in the anterior maxilla.

## Figures and Tables

**Table 1:** Initial clinical and CBCT examination before and 4 months after implant placement and GBR procedure.

	Site	Baseline (mm)	4 Months (mm)	Difference (mm)
Buccal-palatal bone width	21	6.0	7.4	1.4
	12	5.8	7.9	2.1
	14	4.7	7.1	2.4
Gingival Thickness		1.0	2.0	1.0

## Data Availability

Additional data are available upon request to the authors.
